# Deep Breathing Practice Facilitates Retention of Newly Learned Motor Skills

**DOI:** 10.1038/srep37069

**Published:** 2016-11-14

**Authors:** Goldy Yadav, Pratik K. Mutha

**Affiliations:** 1 Centre for Cognitive Science, Indian Institute of Technology Gandhinagar, Gujarat, India; 2 Department of Biological Engineering, Indian Institute of Technology Gandhinagar, Gujarat, India

## Abstract

Paced deep breathing practices, a core component of a number of meditation programs, have been shown to enhance a variety of cognitive functions. However, their effects on complex processes such as memory, and in particular, formation and retention of motor memories, remain unknown. Here we show that a 30-minute session of deep, alternate-nostril breathing remarkably enhances retention of a newly learned motor skill. Healthy humans learned to accurately trace a given path within a fixed time duration. Following learning, one group of subjects (n = 16) underwent the 30-minute breathing practice while another control group (n = 14) rested for the same duration. The breathing-practice group retained the motor skill strikingly better than controls, both immediately after the breathing session and also at 24 hours. These effects were confirmed in another group (n = 10) that rested for 30 minutes post-learning, but practiced breathing after their first retention test; these subjects showed significantly better retention at 24 hours but not 30 minutes. Our results thus uncover for the first time the remarkable facilitatory effects of simple breathing practices on complex functions such as motor memory, and have important implications for sports training and neuromotor rehabilitation in which better retention of learned motor skills is highly desirable.

There is growing scientific interest in understanding the effects of simple, paced, deep breathing practices on cognitive function, and delineating the mechanisms that underlie these effects. These practices, which form an integral component of a number of meditation programs^1^, generally involve inhalation and exhalation of air at a pre-determined rate that is different from the regular breath cycle. Depending on the breathing pattern, these practices can take various forms^2^ – paced uni-nostril breathing (deep breathing through a single nostril at a pace that is different than normal breathing), paced alternate-nostril breathing (paced, deep breathing while switching between the two nostrils, also at a rate different than normal), coherent or resonance breathing (breathing at a rate of 5–6 cycles per minute with identical pace of inhalation and exhalation), or resistance breathing (breathing while creating resistance to the airflow using pursed lips or clenched teeth).

A number of studies suggest that these breathing practices result in marked beneficial effects across a variety of cognitive functions. For instance, Busch *et al*.^3^ demonstrated that relaxed deep slow breathing practice significantly increased detection and pain thresholds for cold and hot stimuli, and also reduced negative emotions. Verbal and spatial performance has also been shown to benefit from uni-nostril breathing practice^4^. Gothe *et al*.^5^ examined the effects of yoga exercises that included meditative deep breathing on executive functions like inhibitory control and working memory. They reported that following this training, performance on flanker and n-back tasks, which were used to assess these functions, was significantly better than baseline levels. In fact, participants’ performance was also better than that observed after a session of aerobic exercise. Similarly, Telles *et al*.^6^ showed improved static motor performance in a group of children who trained on a combination of exercises that included paced breathing practice, postural training, visual focusing exercises and games to improve attention and memory. Performance in a task that required placement of a metal stylus into holes of various sizes was much better in the trained group compared to another that did not undergo the training. Thus, collectively, these studies demonstrate enhanced performance across a variety of cognitive functions when following a regimen that includes deep breathing practices. However, because these studies also generally include other exercises along with the breathing practice, it is difficult to identify whether and how much the breathing practice alone contributes to this enhancement. Additionally, the effects of such respiratory exercises on more complex functions such as memory are not clear. In particular, whether deep breathing practices impact the formation and retention of motor memories, both in the short and the longer term, remains unknown. Understanding whether these simple techniques can modulate motor memories developed through learning of new motor skills is important because they can then potentially be used in conjunction with other methods to promote faster recovery in patients being trained to re-learn motor skills lost as a consequence of neurological injury.

An interaction between motor memory formation and consolidation, and deep breathing practices appears highly likely. Learning and retention of motor skills is supported by a complex neuroanatomical architecture that includes dorsolateral prefrontal cortex, posterior parietal cortex and primary motor cortex, as well as subcortical structures such as basal ganglia and cerebellum^7^,^8^. Interestingly, activity in the same regions of the brain appears to be modulated through voluntary changes in the pattern and the duration of breath^9^,^10^,^11^. This overlap suggests that deep, paced breathing could exert a strong influence on how a motor skill is learned and retained. In this study, we hypothesized that paced deep respiratory exercise following motor skill learning would lead to better retention of that skill. Young, healthy human subjects first learned a new motor task (Fig. 1), then practiced a single 30 minute session of paced, alternate-nostril breathing exercise, and were then tested for retention of the learned skill immediately following the breathing session as well as 24 hours later (BREATHING_IMM group). Performance of this group was compared to CONTROL participants who quietly rested after motor learning for 30 minutes, but were tested for retention at identical time points. We later also recruited a third group of participants who rested post motor learning, but practiced paced breathing after their first retention test (BREATHING_LATE group).

## Results

### Retention of the learned motor skill was better at 30 minutes and at 24 hours in the BREATHING_IMM group

#### Learning Phase

Figure 2 compares the learning performance of the CONTROL (blue) and the BREATHING_IMM (red) groups. As shown, both groups showed large errors initially, but these errors decreased with practice. In fact, by the end of the learning phase (bin 10), errors were almost zero for subjects in both groups. Our group (CONTROL, BREATHING_IMM) × time (first learning bin, last learning bin) ANOVA revealed only a main effect of bin (F_1,28_ = 44.4874, p < 0.0001). No significant main effect of group (F_1,28_ = 0.1019, p = 0.7520) or group X bin interaction (F_1,28_ = 0.0012, p = 0.9723) was noted. This suggested that both groups reduced their motor errors during the learning phase in a similar manner.

#### Retention at 30 minutes

Following the learning phase, participants in the BREATHING_IMM group practiced respiratory breathing exercises for 30 minutes while the CONTROL group rested for the same duration. We then examined whether the learning that occurred during the learning phase would be retained differently in the two groups after their respective 30-minute sessions. To do so, we compared the errors on last bin of the learning phase with those on first bin of the retention session during which participants practiced the same motor task. As shown in Fig. 2, errors in the CONTROL group (blue) were larger on the first bin of the retention session compared to their errors on the last learning bin. In contrast, errors of the BREATHING_IMM group (red) appeared to be similar at both time points. This was statistically confirmed in our group (CONTROL, BREATHING_IMM) × time (last learning bin, first 30-min retention bin) ANOVA, which showed a significant group x time interaction (F_1,28_ = 7.6234, p = 0.0101). Post-hoc analyses revealed clear differences both within and between groups. Errors on the first retention bin were significantly different for the CONTROL group (p = 0.0004), but not the BREATHING_IMM group (p = 0.8031) compared to their respective errors on the last learning bin. More crucially however, we noted a significant difference in the errors for the two groups on the first retention bin (p = 0.0005). The smaller errors in the BREATHING_IMM group indicated that this group had retained their learning better than the CONTROL group. When we examined the errors for the entire retention session, instead of just the first bin, the pattern of results was similar. We found significantly smaller errors for the BREATHING_IMM group compared to the CONTROL group (F_1,28_ = 9.1679, p = 0.0052).

#### Retention at 24 hours

To assess retention of learning in the longer-term, we compared the motor errors on the last learning bin with those on the first bin of the retention test performed 24 hours later for the CONTROL and BREATHING_IMM groups. As can be seen in Fig. 2, the BREATHING_IMM group (red) again showed much smaller errors at 24 hours compared to the CONTROL group (blue). Our group (CONTROL, BREATHING_IMM) × time (last learning bin, first 24 hr. retention bin) ANOVA showed a significant interaction between group and condition (F_1,28_ = 8.4036, p = 0.0072). Post hoc analyses again revealed significant within and between group differences. While errors on the first retention bin were significantly different compared to the last learning bin for the CONTROL participants (p = 0.0014), this was not the case for the BREATHING_IMM group (p = 0.9953). Moreover, the BREATHING_IMM group demonstrated significantly smaller errors on the first bin at 24 hours compared to the CONTROL group (p = 0.0001). When we compared errors between the groups for the entire 24-hour retention session, we found a similar trend. The BREATHING_IMM participants showed much smaller errors compared to the CONTROL participants (F_1,28_ = 8.1506, p = 0.0080). This indicated that the group that practiced the breathing exercise demonstrated better retention of the learned motor skill even 24 hours post learning.

### Retention of the learned motor skill was better at 24 hours in the BREATHING_LATE group

Motivated by the findings in the BREATHING_IMM group, we recruited a third group of participants (BREATHING_LATE); subjects in this group first learned the same motor skill, and then rested for 30 minutes like the CONTROL group. They were then tested for retention by examining performance on the same motor task. Following this session, subjects in the BREATHING_LATE group practiced the same breathing exercise as the BREATHING_IMM group for 30 minutes. We then examined retention of the learned motor skill 24 hours later. We predicted that learning in the BREATHING_LATE group would be similar to the other two groups. However, we expected that retention in this group at 30 minutes would be closer to the CONTROL group. Further, since this group would have practiced the breathing exercise following this 30-min test session, they would demonstrate better skill retention 24 hours later.

#### Learning Phase

As expected, learning of the motor skill was not different between the BREATHING_LATE (green) and the other two groups (Fig. 2). Our group (CONTROL, BREATHING_IMM, BREATHING_LATE) X time (first learning bin, last learning bin) ANOVA revealed neither a significant effect of group (F_2,37_ = 0.0673, p = 0.9350) nor a significant interaction between group and bin (F_2,37_ = 0.0561, p = 0.9455). Only a significant effect of bin was noted (F_1,37_ = 69.8124, p < 0.0001), which indicated that motor errors decreased with practice. Thus, subjects in the BREATHING_LATE group learned to a similar extent as the CONTROL and BREATHING_IMM groups.

#### Retention at 30 minutes

We then examined how well the BREATHING_LATE group retained the learned motor skill after 30 minutes of rest. As shown in Fig. 2, similar to the CONTROL group (blue), retention at 30 minutes was poor in the BREATHING_LATE group (green). The group (CONTROL, BREATHING_IMM, BREATHING_LATE) X time (last learning bin, first 30 min retention bin) ANOVA showed a significant interaction (F_2,37_ = 6.0262, p = 0.0054), with post-hoc tests showing that motor errors on bin 1 of the retention session were similar in the CONTROL and BREATHING_LATE groups (p = 0.9955). Further, the errors in the BREATHING_LATE group (green) were greater than those seen for the BREATHING_IMM group (red) (p = 0.0009). Thus, when the BREATHING_LATE group had not performed the breathing exercise, their performance was similar to the CONTROL group. This was true even when we considered the entire retention session for our analysis. We found a significant effect of group in a one-way ANOVA (F_2,37_ = 3.9216, p = 0.0285), with post-hoc tests showing smaller errors in BREATHING_IMM group compared to both the CONTROL (p = 0.0251) and the BREATHING_LATE (p = 0.0221) groups. Additionally, there was no difference between the CONTROL and the BREATHING_LATE groups (p = 0.7943).

#### Retention at 24 hours

Subjects in the BREATHING_LATE group practiced the breathing exercise after their first retention test session, and were then re-tested on the motor task 24 hours later. Remarkably, this group now showed near perfect retention of the motor skill. Their errors on the first bin of the 24-hour retention test were close to zero (Fig. 2). Our group (CONTROL, BREATHING_IMM, BREATHING_LATE) X time (last learning bin, first 24 hr retention bin) ANOVA again showed a significant interaction (F_2,37_ = 5.7388, p = 0.0067). Post-hoc tests indicated that motor errors of the BREATHING_LATE group (green) on the first bin of the 24-hour retention test were not significantly different from their errors on the last bin of the learning phase (p = 1.0000). We separately confirmed that these errors were also not significantly different from their errors on the last bin of the 30-minute retention test (p = 0.8743). Crucially, their errors on the first bin at 24 hours post the breathing exercise were significantly smaller than those of the CONTROL group (blue) (p = 0.0003), but were not different from those of the BREATHING_IMM group (red) (p = 1.0000). This suggested that following the breathing exercise, retention at 24 hours improved significantly in the BREATHING_LATE group to a level that was not different from the group that performed the breathing exercise right after motor skill learning. When we considered the entire 24 hour retention session, group differences did not reach statistical significance (F_2,37_ = 2.6081, p = 0.0872). In general however, retention, when assessed over an entire session that also involves additional practice, must be interpreted with caution because performance beyond the first few trials also includes the effects of re-learning. Early performance, such as that seen during the first bin in our case for example, may be a better indicator of retention in such cases.

## Discussion

We tested whether paced deep breathing practice performed immediately after learning a new motor skill has an impact on how well that skill is retained. Our findings indicated that a single 30-minute session of deep alternate-nostril breathing significantly facilitated the retention of a newly acquired motor skill. Better retention was evident immediately, as well as 24 hours after breathing practice, suggesting that such simple practices can be exploited as an effective tool to enhance both short term and somewhat longer term retention of skilled movements. These results contribute significantly to research exploring factors that promote motor skill learning and retention, as well as studies examining the impact of deep breathing practices on cognitive function.

Numerous studies have proposed that acquisition and retention of motor skills can be influenced by a variety of factors that are both informational and motivational. For example, simply observing subjects learning to reach in a novel environment leads to better performance when subjects are tested later in the same environment^12^. Similarly, feedback indicating better than average performance has been shown to carry motivational value, which then enhances learning^13^. Other factors such as practice structure^14^, sleep^15^ and non-invasive cortical stimulation^16^ have all shown to facilitate learning and retention of motor skills. Some recent work also supports the idea that aerobic exercise can significantly facilitate both learning and retention of motor skills. While moderate exercise appears to aid encoding of motor memories and facilitate learning^17^, high intensity aerobic exercise also improves retention^18^. Our study adds to this growing body of literature aimed at identifying factors that can promote skill learning and retention, and is perhaps the first demonstration that much simpler exercises that involve modulation of breath patterns can also impact retention of newly learned motor skills. Future research may examine the impact of varying the duration, intensity and periodicity of such practices on the degree of retention.

Our findings also add to the range of functions that appear to be affected by deep breathing practices. Block *et al*.^19^ showed that unilateral nostril breathing led to better performance on both spatial and verbal tasks, a result subsequently corroborated by other studies^4^,^20^. Busch *et al*.^3^ demonstrated greater thresholds for pain, reduced sympathetic nervous system activity as measured through lower skin conductance and elevated mood following relaxing deep slow-breathing practice. The influence of such practices on the motor system was examined by Telles *et al*.^6^, who found improved static motor performance in children who practiced an exercise regimen that included deep breathing practice. Our current study suggests that more complex motor functions, including motor memory, can be positively influenced by such practices. This has important implications for sports training, in which retention of newly learned motor skills is highly desirable.

The finding that a simple intervention such as paced breathing may have an effect on motor memory retention may not appear intuitive. However, there are a number of possible explanations for this. Psychological factors such as a calm but alert state of mind^21^, and enhanced self-control and concentration^6^ induced by interventions that involve breathing exercises could result in better performance and retention of the motor task, just as these enhance other cognitive functions. A neurophysiological basis for better retention of motor skills following breathing practice is also likely. A number of reports clearly indicate that deep, paced breathing has an excitatory effect on the vagus nerve^1^,^22^. In animal models, increased vagus nerve stimulation has been demonstrated to improve motor skill acquisition and retention^23^, suggesting that it may promote some of the neuroplastic changes associated with motor training. Such changes are thought to occur via the release of neurochemicals such as acetylcholine, epinephrine and also brain derived neurotrophic factor (BDNF)^24^,^25^,^26^,^27^. Greater levels of these substances, particularly BDNF, have been associated with neurogenesis, neural plasticity and neural repair^28^,^29^. Specifically relevant to our study, Vaynman *et al*.^30^ and Gomez *et al*.^31^ have implicated BDNF in the consolidation of learning in rats. These authors found that spatial learning and retention on the Morris water maze task was better in rats that underwent a weeklong exercise program compared to sedentary controls. When BDNF receptors in the hippocampus were blocked, the exercise-induced benefit was lost. Similarly, studies in humans that have examined the impact of exercise on cognition and motor memory retention have also pointed to BDNF as a potential mediator of enhancement in these functions^32^,^33^. It is thus possible that the greater skill retention seen in our breathing group occurred through breathing-induced stimulation of the vagus nerve, which ultimately led to elevated levels of neurochemical substances like BDNF that facilitate motor memory consolidation. This can be tested in future studies aimed at identifying specific biomarkers associated with breathing practices.

A striking finding in our study was the significantly better retention of the learned motor skill even at 24 hours in the breathing groups. This is non-trivial because benefits seen shortly after learning do not always translate to the longer term^34^, and the effects at these two time frames can indeed be the opposite depending on activity during the intervening period^16^,^35^,^36^. This finding is also important because delayed retention tests are often better signatures of actual learning than short-term changes in performance. In the BREATHING_IMM group, the greater retention at 24 hours could be viewed as a consequence of the additional post-breathing “training” on the motor task during the first retention session. However, this is unlikely because significantly greater retention at 24 hours was also seen in the BREATHING_LATE group, which received no additional “training” post breathing practice. In fact, there was no difference between the two breathing groups in motor error magnitude on the first bin of the 24 hour re-test, suggesting that the additional motor practice that the BREATHING_IMM group received following the breathing session provided no extra benefit in terms of how well the learned motor skill was retained at 24 hours. Rather, our findings suggest that the effects of the deep breathing practice were maintained between days. These results earnestly suggest that a single deep breathing practice session can produce lasting benefits in terms of motor memory retention. This is surprising because such lasting enhancements depend on a number of factors, not all of which were controlled in the current study. Nevertheless, our results suggest that the breathing exercise triggered mechanisms that facilitate long-term consolidation of motor memory. These results mirror those of Roig *et al*.^18^ who found greater retention of a motor skill even at one week post training when motor practice was immediately followed by a high intensity bout of aerobic exercise. A follow-up study by these authors^33^ pointed to greater post-exercise levels of BDNF, norepinephrine and lactate in the circulation, which correlated with better long-term retention of the motor skill. It remains to be seen whether similar biomarkers underlie long term memory of the motor skill following the much simpler breathing exercises explored in the current study, and also whether each biomarker plays a distinct role during this process of motor memory consolidation.

Finally, our results open up the possibility of incorporating simple breathing practices into neurorehabilitation paradigms. Such regimens invariably provide motor (re)training, and emphasize learning and retention of movements lost due to neurological injury. Our results indicate that retention is better when followed by a simple deep breathing session practiced only for 30 minutes after motor training. Given the ease of performing such exercises, they can easily be incorporated into rehabilitation protocols to supplement conventional approaches aimed at facilitating retention of motor training in patients. In fact, the efficacy of such breathing practices towards alleviating some symptoms of Parkinson’s disease^37^, epilepsy^38^, post traumatic stress disorder^22^,^39^, depression^40^, hypertension^41^ and chronic pain^3^, is already being explored and early results show great promise. Our results suggest that such simple deep breathing practices can also be included in rehabilitation of *motor* disability. However, certainty on this front will require more studies that examine performance across a range of motor tasks using a variety of breathing practices in much larger samples. Our current sample size was somewhat small, our motor task was simple and could be learned fairly quickly, the control group was not engaged in a task that was more active (but did not modify breathing rate) and we tested only healthy individuals. Additionally, because we wished to confirm our initial findings, participants that practiced breathing after the first retention test were all assigned to the same group and were not randomized. Future studies may avoid some of these potential limitations and more exhaustively test the influence of paced, deep breathing practices on motor skill retention. Our current results fittingly set the stage to do so.

## Methods

### Ethics Statement

This study was approved by the Institutional Ethics Committee of Indian Institute of Technology Gandhinagar, and was carried out in accordance with the approved guidelines. Participants provided informed consent prior to participation.

### Participants

We recruited a total of 40 right-handed participants (31 Males and 9 Females) in the age group of 18–27 yrs. These were either undergraduate or graduate students at the Indian Institute of Technology Gandhinagar. All subjects were right-handed; handedness was established using the Edinburgh Handedness Inventory^42^. They reported no peripheral movement restriction, orthopedic injury or any history of neurological or psychiatric ailments. They also reported no prior experience with breathing or meditative practices. All subjects were paid for their participation at the end of the experiment.

### Experimental Setup and Task

Participants sat facing a horizontally mounted computer screen that was positioned above a digitizing tablet. They made planar movements by moving a hand-held stylus on the tablet. Direct visual feedback of the hand was not available; it was blocked by the computer screen. Instead, visual feedback about stylus position (and thereby, hand position) was given by means of an on-screen cursor. The position of the cursor was always veridical with respect to hand position. This arrangement was used primarily in order to restrict hand movement to the same dimensions as the visual display, and to ensure that the movement and its visual feedback were in similar planes. The stylus-tablet setup also ensured that subjects would not have to maintain their hands in the air while moving, thereby avoiding the undesirable conditions of not being able to account for motion perpendicular to the display as well as fatigue of the participants.

**Figure 1:**
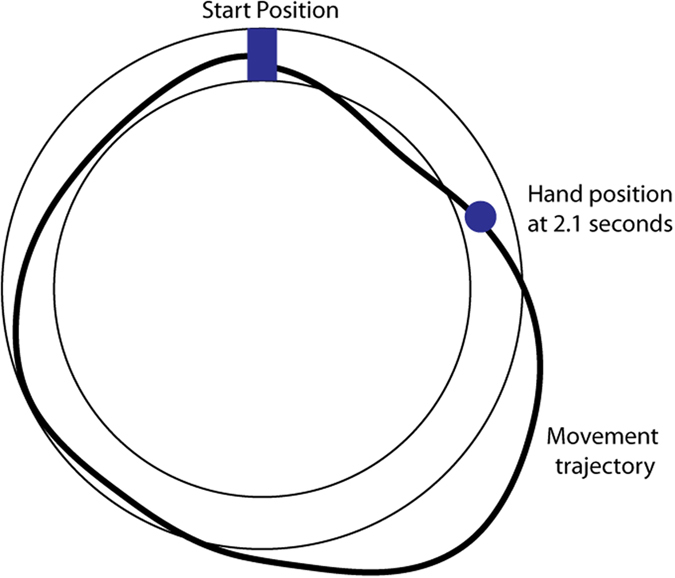
Experimental Task. Subjects learned to trace a circular path in a fixed amount of time without any errors. The start box was shown as a blue rectangle while the location of the hand at 2.1 seconds was shown as a blue disk. Subjects were encouraged to bring this disc in the start box at the end of the movement without crossing the edges of the displayed circular path, essentially requiring them to accurately trace the complete circle within that time frame. A representative trajectory during early learning is shown in black.

**Figure 2:**
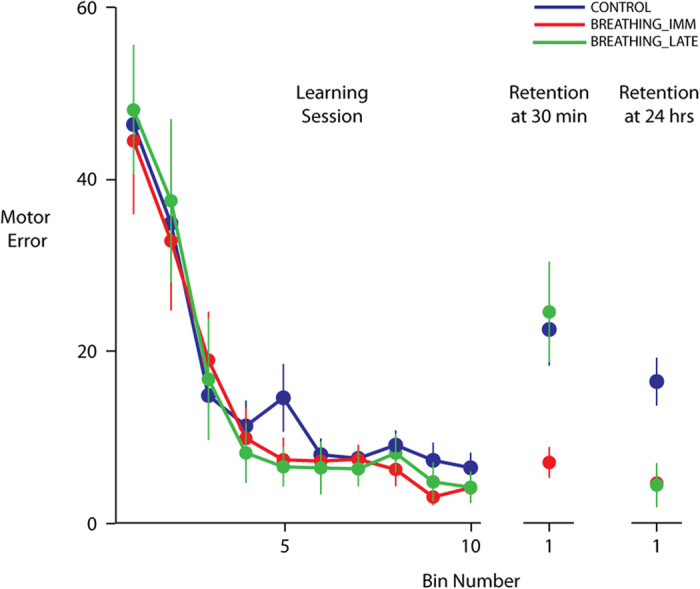
Learning and retention performance of all three groups. Control subjects are shown in blue, while the BREATHING_IMM group and the BREATHING_LATE groups are shown in red and green respectively. All groups learned in a similar manner (left panel). However, clear group differences were seen in the 30 minute and 24 hour retention test sessions. Data shown are mean ± SEM.

On each trial, a rectangular start box (1.3 × 0.57 cm) was displayed at the center of the computer screen. Subjects were required to position their hand (cursor) into this start box to initiate a trial. As soon as subjects entered into the start box, a circular ring (inner radius of 5.69 cm and outer radius of 7.12 cm) circumscribing it was displayed on the screen (Fig. 1). Once subjects had stayed in the start box for 100 msec, an audiovisual go signal, which comprised of a change in color of the start box and an auditory tone, was delivered; this served as a cue for participants to initiate the required movement. Subjects were required to learn to move their hand within the circular ring without crossing its edges and complete the full circular movement back to the start box within 2.1 seconds. Cursor feedback was available throughout the movement, and in addition, a circular disk was displayed at the location of the hand at 2.1 seconds (Fig. 1). Subjects were encouraged to bring this disc in the start box at the end of the movement without crossing the edges of the displayed ring, essentially requiring them to accurately trace the complete circle within the specified timeframe. Subjects thus had to learn to be fast and accurate at the same time, a key aspect of motor skill learning. At the end of each trial, subjects were shown the trajectory they had traced and were also given feedback about their movement errors. This feedback was a numerical score that comprised of both, spatial errors in keeping within the bounds of the ring as well as a failure to complete tracing within the stipulated time of 2.1 seconds. Importantly, the disc (hand location at 2.1 seconds) allowed us to translate the timing errors into spatial errors. The numerical error score was simply the sum of these two spatial errors (in arbitrary units) and subjects were encouraged to keep this value as low as possible.

Subjects performed 100 such tracing trials in which they learned to accurately trace the ring in the given amount of time. Following this learning block, they were randomly divided using simple randomization procedures into two groups, CONTROL (n = 14) and BREATHING_IMM (n = 16). The BREATHING_IMM group practiced deep alternate-nostril breathing (see below) at the rate of 8–10 breath cycles per minute for 30 minutes, while participants in the CONTROL group were asked to sit and relax in a quiet room with no distractors. Following these sessions, both groups were tested for retention of the learned motor skill in which they again performed the same motor task for 100 trials. A similar test of retention was performed after 24 hours. The results of this experiment showed better retention of the learned motor skill in the BREATHING_IMM group, both immediately and at 24 hours post-learning. To further confirm these effects, and to rule out other factors such as extended practice, we recruited a third group of participants (BREATHING_LATE, n = 10) who rested quietly for 30 minutes after the learning session (similar to the CONTROL group), were then tested for skill retention, and then practiced the paced breathing exercise for 30 minutes. This group was then tested for retention of the motor skill again at 24 hours.

### Deep Breathing Practice

The BREATHING_IMM and BREATHING_LATE groups performed alternate-nostril breathing exercises for 30 minutes. The number of breath cycles per minute was fixed at 8–10 cycles, as is generally prescribed in paradigms that employ such practices^21^,^43^. Participants were seated on the floor in a crossed-legged posture with spine erect and were instructed to block one of their nostrils with the thumb and inhale slowly through the other nostril. They were asked to rest the other hand on their knee. Following inhalation, subjects held their breath for 2 seconds and then exhaled slowly through the other nostril while closing the nostril through which they had previously inhaled. Next, they were instructed to inhale through the currently open nostril and exhale through the other. Thus, each nostril was alternately used for inhalation and exhalation. Subjects were instructed and encouraged to maintain a fixed pattern and duration of 1:1:2 for inhalation, hold and exhalation. In other words, they were required to inhale for 2 seconds, hold their breath for 2 seconds and then exhale over 4 seconds. For this, they were guided by a tone and were monitored by an experimenter.

### Data Analysis

Our primary measure of interest was motor error during the tracing. Our choice of this variable was motivated by prior studies that have emphasized improvements in motor accuracy as a measure of motor skill learning^17^,^18^,^44^. In line with these studies, we examined the reduction in motor errors with practice as an indicator of skill learning. Trials were divided into bins, with each bin comprising of 10 trials, and the mean motor error for each bin was calculated. Trials in which movement time exceeded 4 seconds were excluded from these calculations (3.825% of all trials). To examine learning and retention, we subjected this error to a two-way analysis of variance with group and bin as factors. Tukey’s post hoc tests were conducted when warranted by significant effects in the ANOVA.

## Additional Information

**How to cite this article**: Yadav, G. and Mutha, P. K. Deep Breathing Practice Facilitates Retention of Newly Learned Motor Skills. *Sci. Rep.* **6**, 37069; doi: 10.1038/srep37069 (2016).

**Publisher's note:** Springer Nature remains neutral with regard to jurisdictional claims in published maps and institutional affiliations.
